# The ABA–AtNAP–SAG113 PP2C module regulates leaf senescence by dephoshorylating SAG114 SnRK3.25 in Arabidopsis

**DOI:** 10.1186/s43897-023-00072-1

**Published:** 2023-10-30

**Authors:** Gaopeng Wang, Xingwang Liu, Su-Sheng Gan

**Affiliations:** 1 Present Address: Shanghai Institute of Technology, Shanghai, 201418 China; 2 Present Address: Beijing Key Laboratory of Growth and Developmental Regulation for Protected Vegetable Crops, College of Horticulture, China Agricultural University, Beijing, 100193 China; 3 Plant Biology Section, School of Integrative Plant Science, Cornell University, Ithaca, NY 14853 USA

**Keywords:** Aging, Leaf senescence, Phosphorylation, Protein phosphatase, Sucrose nonfermenting 1-related kinase

## Abstract

We previously reported that ABA inhibits stomatal closure through AtNAP-SAG113 PP2C regulatory module during leaf senescence. The mechanism by which this module exerts its function is unknown. Here we report the identification and functional analysis of *SAG114*, a direct target of the regulatory module. *SAG114* encodes SnRK3.25. Both bimolecular fluorescence complementation (BiFC) and yeast two-hybrid assays show that SAG113 PP2C physically interacts with SAG114 SnRK3.25. Biochemically the SAG113 PP2C dephosphorylates SAG114 in vitro and *in planta*. RT-PCR and GUS reporter analyses show that *SAG114* is specifically expressed in senescing leaves in Arabidopsis. Functionally, the SAG114 knockout mutant plants have a significantly bigger stomatal aperture and a much faster water loss rate in senescing leaves than those of wild type, and display a precocious senescence phenotype. The premature senescence phenotype of *sag114* is epistatic to *sag113* (that exhibits a remarkable delay in leaf senescence) because the *sag113 sag114* double mutant plants show an early leaf senescence phenotype, similar to that of *sag114*. These results not only demonstrate that the ABA-AtNAP-SAG113 PP2C regulatory module controls leaf longevity by dephosphorylating SAG114 kinase, but also reveal the involvement of the SnRK3 family gene in stomatal movement and water loss during leaf senescence.

**Graphical Abstract:**

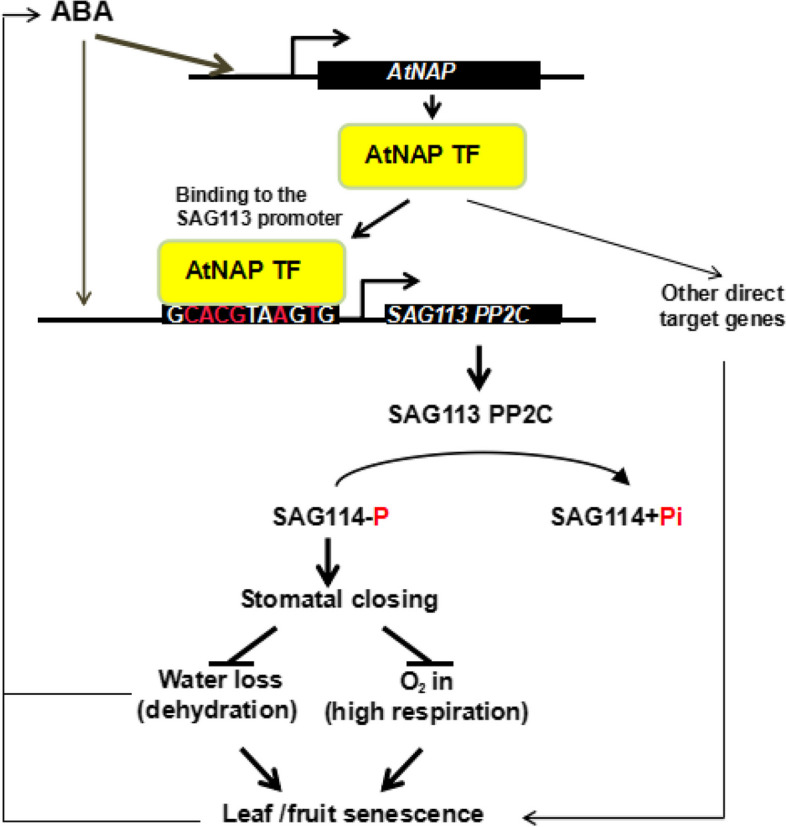

**Supplementary Information:**

The online version contains supplementary material available at 10.1186/s43897-023-00072-1.

## Core

*SAG114* encodes for SnRK3.25, and is the direct target of the ABA-AtNAP transcription factor-SAG113 PP2C regulatory module; the module dephosphorylates the SAG114 kinase to control leaf senescence in Arabidopsis.

## Gene and accession numbers

*AtNAP*, At1G69490; *SAG113*, At5G59220; *SAG114*, At5G25110

## Introduction

Leaf senescence is an age-associated developmental process involving ordered dismantlement of subcellular structures, degradation of (macro)molecules such as chlorophyll, proteins, lipids, DNA and RNA, and recycling of the released nutrients to seeds, storage organs and/or actively growing tissues (Guo et al. 2004; Guo 2013; Hortensteiner 2013; Takami et al. 2018; Anna et al. 2019; Guo et al. 2021; Cao et al. 2022; Gan 2022). In a natural setting, plants are frequently exposed to unfavorable environmental conditions and these abiotic and biotic stresses can readily induce leaf senescence. It is generally accepted that age-dependent and/or stress-induced leaf senescence is driven by massive differential gene expression, especially those senescence-associated genes (*SAG*s) that are upregulated at the onset of and during senescence. The activation of these *SAG*s is achieved by transcription factors that bind to specific nucleic acid sequences of *SAG* promoters to cause RNA polymerase II to transcribe the genes (Guo and Gan 2014). Transcription factors (TFs) such as NAC, WRKY, MYB and bZIP are reported to be vital regulators of the *SAG* expression (Guo et al. 2004; Miao et al. 2004; Balazadeh et al. 2010; Janack et al. 2016; Liu et al. 2016; Li et al. 2017; Jia et al. 2019; Cao et al. 2023).

AtNAP, a NAC family transcription factor, plays a pivotal role in senescence of leaves and carpels in Arabidopsis (Guo and Gan 2006; Kou et al. 2012). Its orthologue genes in rice, cotton and maize are also shown to have a key role in leaf senescence (Zhang et al. 2012b; Liang et al. 2014; Fan et al. 2015). *NAP*s are highly regulated by ABA (Zhang and Gan 2012; Liang et al. 2014), and several direct target genes have been identified (Zhang and Gan 2012; Hu et al. 2021; Wang et al. 2022). One of the direct target genes is *SAG113* (Zhang and Gan 2012), which encodes a protein phosphatase 2C (PP2C) that prevents stomata from closing at the onset of and during leaf senescence, such that enough oxygen can get into the mesophyll cells for surging respiration needs, and at the same time, water can be transpired more easily via the open stomata to facilitate senescence (Zhang and Gan 2012; Zhang et al. 2012a). The regulatory mechanisms underlying the ABA-AtNAP-SAG113 PP2C module remain to be deciphered.

There are more than 180 genes whose products may be involved in signal transductions during leaf senescence in Arabidopsis (Guo et al. 2004; Cao et al. 2022), and protein kinases and phosphatases are important components of the signal transduction systems. Among the protein kinases are mitogen-activated protein kinases (MAPKS), calcium-dependent protein kinases (CDPKs) and most of the SNF (sucrose non-fermenting)1-relasted kinases (SnRKs) (Zhou et al. 2009; Kulik et al. 2011). The SnRKs are a class of serine/threonine protein kinases that belong to the AMPK (adenosine monophosphate-activated protein kinase)-related superfamily in eukaryotes. They are involved in a variety of signaling pathways and play a pivotal role in plant growth and stress responses in plants (Coello et al. 2010; Jamsheer et al. 2019). SAG114, encoding SnRK3.25/CIPK25 (calcineurin β-like interacting protein kinase), is a typical gene in SnRK family, which may possess two main regions: Ser/Thr kinase domain and NAF domain (Fig. S1). The N-terminal region of SAG114 comprises a conserved catalytic domain typical of Ser/Thr kinase, and functions mainly in protein phosphorylation to control the activity of this protein. In contrast, the much less conserved C-terminal domain appears to be unique to this subgroup of kinases. The only exception is the NAF domain that forms an ‘island of conservation’ in this otherwise variable region. The NAF domain has been named after the prominent conserved amino acids Asn-Ala-Phe. It represents a minimum protein interaction module that is both necessary and sufficient to mediate the interaction with the CBL calcium sensor proteins. Plant SnRKs have different roles in different plants such as Arabidopsis, rice and maize, and almost all of them are related with osmotic stress (Halford and Hey 2009).

Here, we report the identification and functional analysis of SAG114 SnRK3.25 that is dephosphorylated by the ABA-AtNAP-SAG113 PP2C regulatory module and controls leaf senescence in Arabidopsis.

## Results

### *SAG114 SnRK3.25* was specifically expressed in senescing leaves

**Fig. 1:**
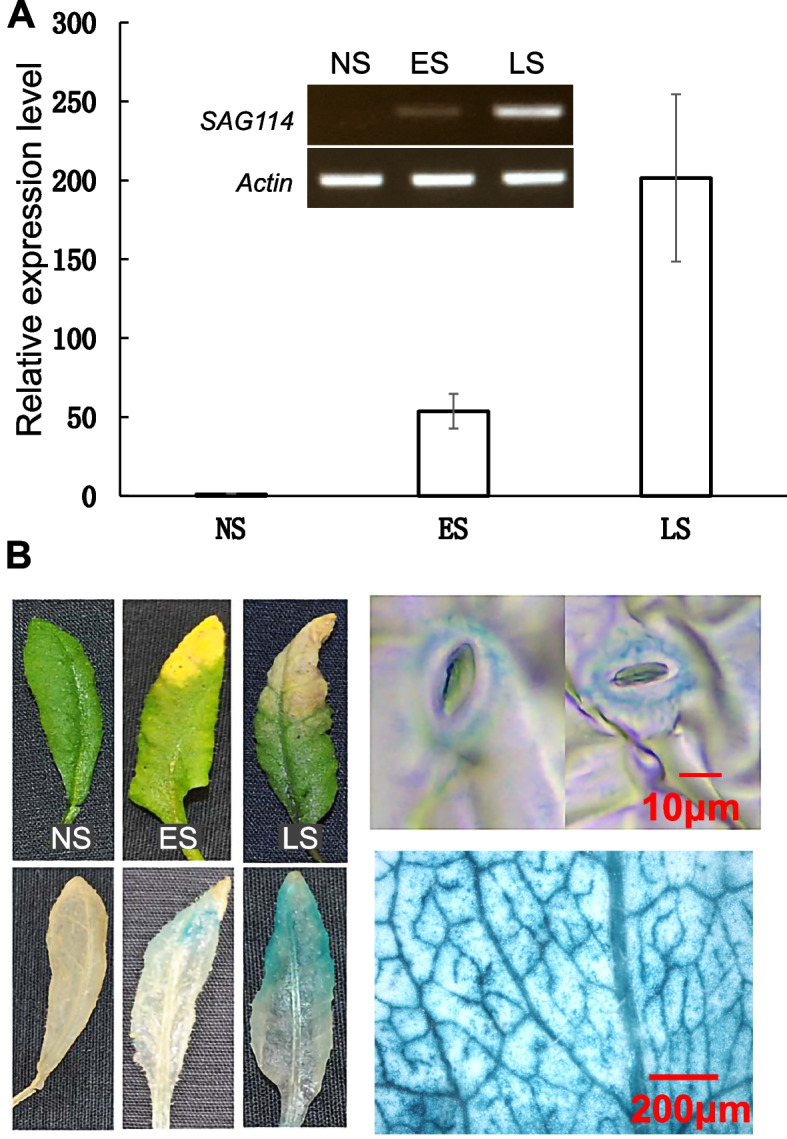
The leaf senescence-specific expression of *SAG114 SnRK3.25* in Arabidopsis. **A** Relative expression levels of *SAG114* in leaves at different stage in Arabidopsis revealed by qPCR analysis. NS, fully expanded non-senescent leaves; ES, early senescence with up to 25% leaf yellowing; LS, late senescence with > 50% leaf yellowing. The data are presented as the means ± SE (n ≥ 6). Insert represents the semi-quantitative PCR product of the *SAG114* transcripts with 28 cycles. **B** GUS staining of leaves of the P_*SAG114*_-*GUS* transgenic plants. The two panels on the right are close-ups of the GUS stained leaves and stomata

We previously established an Arabidopsis leaf senescence transcriptome that represents ~ 2500 *SAG*s (Guo et al. 2004). Among them was *SAG114* that encodes SnRK3.25 (At5G25110), a member of the serine/threonine protein kinase superfamily (Fig. S1) (Hrabak et al. 2003). Both RT-PCR and qRT-PCR analyses revealed that the transcripts of *SAG114* were hardly detectable in fully expanded non-senescing leaves (NS), and that the transcript levels increased with the progression of leaf senescence (Fig. 1A). The *SAG114* promoter (P_*SAG114*_) was used to direct the GUS reporter gene expression, and the GUS staining was shown in the senescent parts of the leaves only (Fig. 1B), which confirmed the senescence-specific expression of *SAG114*. The *GUS* staining also revealed that *SAG114* was highly expressed in veins and guard cells of the senescing leaves (Fig. 1B).

### The *sag114* null mutants exhibited early leaf senescence and fast water loss phenotypes

**Fig. 2:**
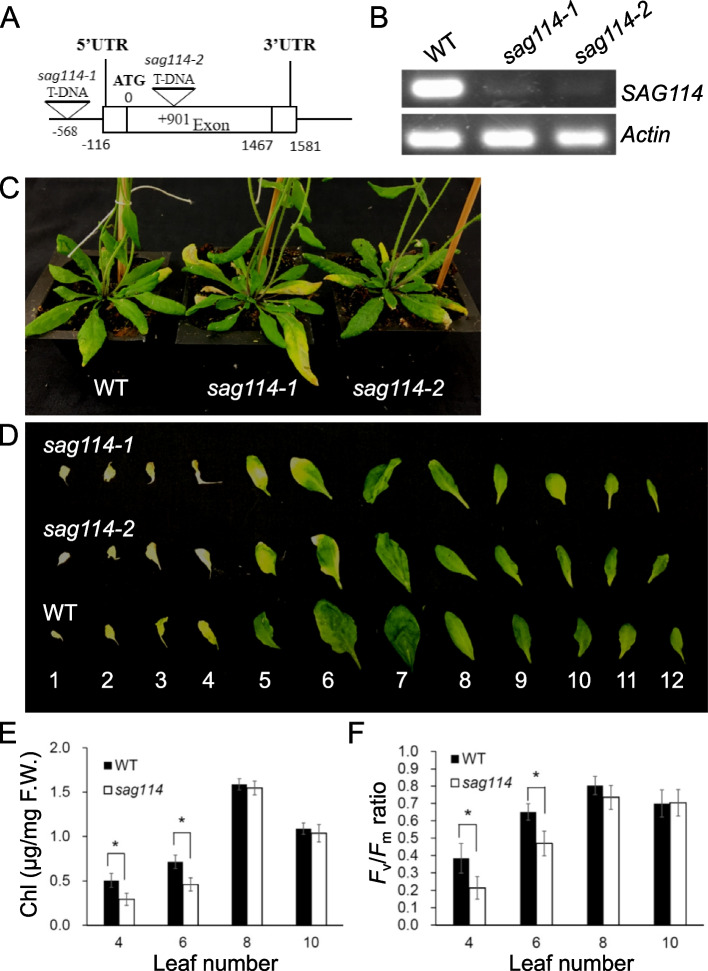
Molecular and functional analyses of *SAG114 SnRK3.25* in leaf senescence. **A** Diagram of *SAG114* gene structure and T-DNA insertion sites. There was no intron in the gene. The Arabidopsis T-DNA line SALK_060162 was designated as *sag114-1,* and SALK_079011 as *sag114-2.* **B** RT-PCR analysis of the *SAG114* expression in senescing leaves of wild-type (WT), *sag114-1* and *sag114-2* mutant plants. **C** Phenotypes of age-matched WT, *sag114-1* and *sag114-2* plants. **D** Alignment of age-matched rosette leaves detached from the respective plants (the leaves were counted from bottom). Both *sag114-1* and *sag114-2* null mutants showed almost the same early leaf senescence phenotype, and only *sag114* designation will be used for hereafter analyses. The chlorophyll (Chl) contents (**E**) and the *F*_v_*/F*_m_ ratios (**F**) in leaves of WT and two *sag114* null mutants. Mean values of four samples ± se are shown (n ≥ 3). Asterisks indicate significant differences between wild-type and transgenic plants (Student’s *t* test, *P* < 0.05)

To investigate the biological function of *SAG114*, we obtained two Arabidopsis lines with T-DNA insertion in the promoter (SALK_060162; desinated as *sag114-1*) and in the coding region (SALK_079011; *sag114-2*), respectively (Fig. 2A). The T-DNA insertion diminished the expression of *SAG114* in these lines (Fig. 2B). Both null mutants displayed a precocious leaf senescence phenotype compared with WT (Fig. 2C and D), and the growth and development prior to the onset of senescence were not distinguishable among the mutants and WT. The chlorophyll concentrations and *F*_v_*/F*_m_ ratios were significantly lower in the 4^th^ and 6^th^ leaves of *sag114* mutants than those in the age-matched leaves of WT (Fig. 2E and F), which was consistent with the early leaf senescence phenotype in *sag114*. The *F*_v_*/F*_m_ ratio is an indicator of the photosystem II activity, and a non-senescing leaf has a ratio of ~ 7 (Hu et al. 2021).

**Fig. 3:**
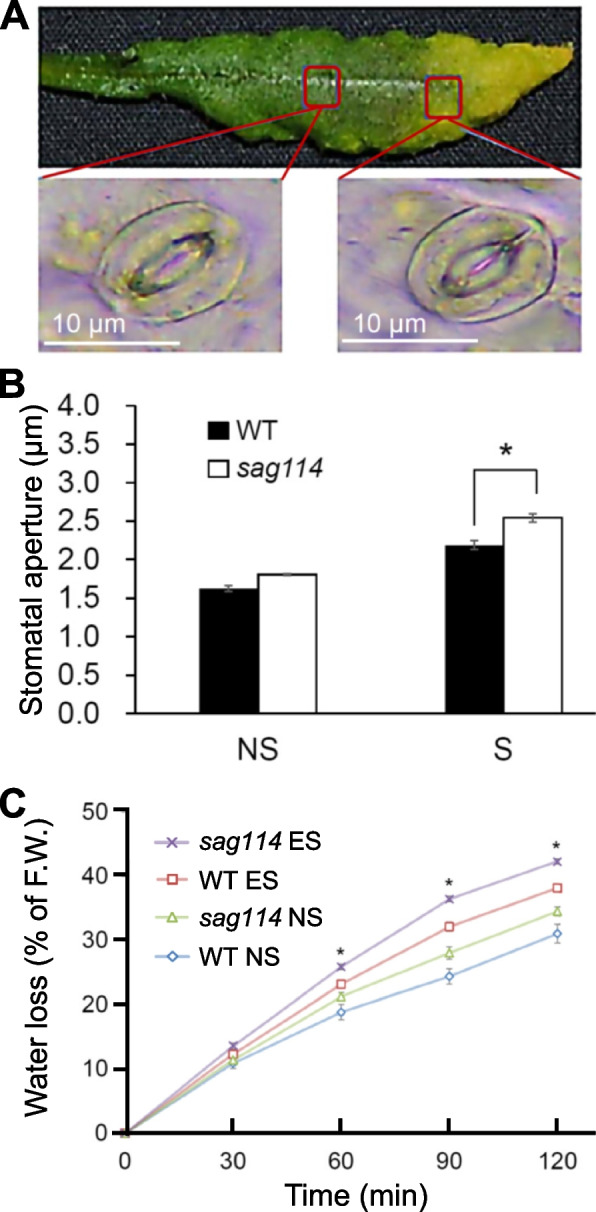
Larger stomatal aperture and faster water loss in leaves of *sag114* null mutant compared with WT. **A** Example of a senescing leaf of *sag114* showing a pair of guard cells with larger stomatal aperture in senescent part and smaller aperture in non-senescent part of the leaf. **B** Significantly larger aperture in senescent leaves of *sag114* than WT. NS, non-senescent leaves without any yellowing; S, senescent leaves that are fully yellowed. **C** Faster water loss in *sag114.* Asterisks indicate significant differences between WT and *sag114* plants (Student’s *t* test, *P* < 0.05)

Considering *SAG114* was expressed in guard cells (Fig. 1B) and the loss-of-function of the gene might alter the stomatal movement, we thus measured the stomatal apertures in non-senescing and senescent parts of leaves in WT and *sag114* null mutants, respectively. The stomatal aperture in senescent part of a leaf in WT was larger than that in non-senescent part of the leaf (Fig. 3A and B). In contrast, the stomatal aperture in senescent part of the *sag114* leaves was significantly larger than that of WT (Fig. 3B). The stomatal apertures in non-senescent parts appeared to be larger in sag114 than WT but the difference was not significant statistically (Fig. 3B). Consistent with differences in the stomatal apertures, the *sag114* leaves lost water much faster than the WT leaves did (Fig. 3C).

### SAG114 was localized in Golgi apparatus

**Fig. 4:**
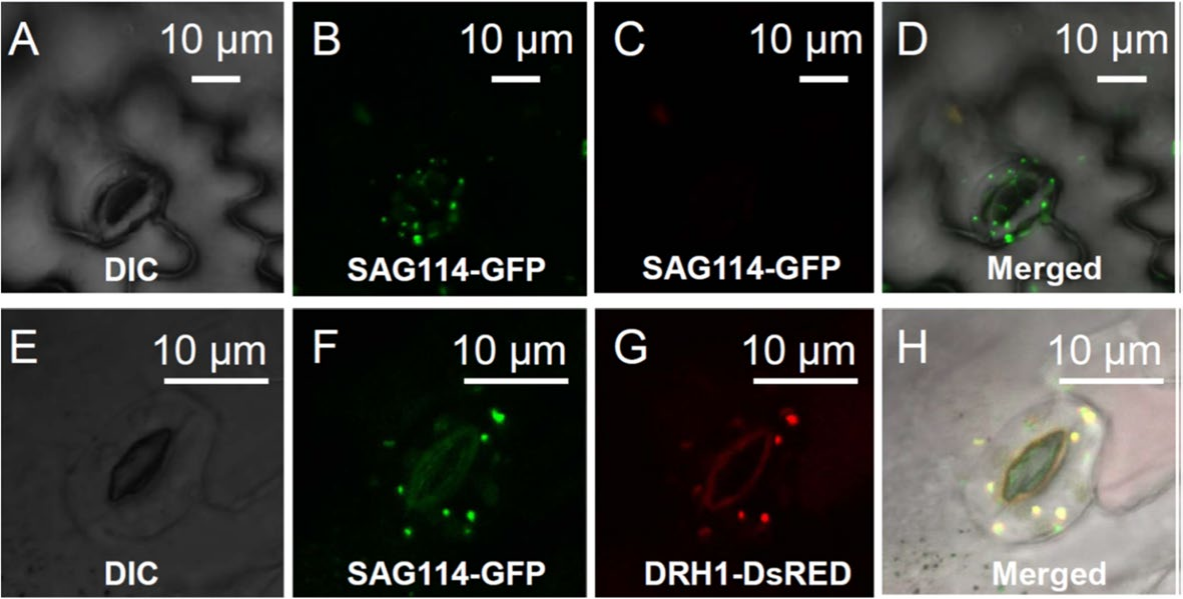
Localization of SAG114 SnRK3.25 in the Golgi apparatus. **A** Differential interference contrast (DIC) image of the epidermis of a transgenic plant expressing GFP under the direction of *SAG114* promoter  (as a control). **B** The GFP expression in A imaged using the eGFP channel setting of Leica DM5500. **C** No GFP signal shown in B could be imaged using the DsRED channel setting of Leica DM5500. **D** Merged image of A-C showing that the SAG114-GFP fusion protein localized to the Golgi apparatus and/or mitochondria. **E** Differential interference contrast (DIC) image of a senescing leaf epidermis of a transgenic plant containing GFP-tagged SAG114 (SAG114-GFP) and a Golgi marker DsRFP-tagged ERH1 (ERH1-DsRED). **F** The GFP expression in the guard cells shown in E imaged using the eGFP channel setting of a confocal microscope (Leica DM5500). **G** Red fluorescent protein expression in the guard cells shown in E taken using the DsRED channel setting of the confocal microscope (Leica DM5500). **H** Merged image of E–G showing that the SAG114-GFP fusion protein co-localized with the *cis*-Golgi marker ERH1-DsRED

To understand the subcellular mechanism underlying *SAG114*, we fused the *SAG114* full-length coding sequence with the *GFP* coding sequence and examined the fusion protein’s subcellular localization using a confocal microscope. The green fluorescence signal was observed in small subcellular vesicles in the guard cells (Fig. 4B and D). The vesicles could be the Golgi apparatus and/or mitochondria, among others. To further determine the precise location of SAG114, a known Golgi marker, ERH1-DsRed (Wang et al. 2008; Zhang et al. 2012a), was transferred into the SAG114-GFP transgenic plants and imaged using the DsRED channel setting of the confocal microscope (Fig. 4G). The red fluorescence signal from the Golgi marker completely overlapped with the green fluorescence signal from the SAG114-GFP fusion protein in the same cells (Fig. 4F, G and H). These data strongly suggested that the SAG114 protein should be in the Golgi apparatus.

### SAG114 physically interacted with SAG113 PP2C in yeast cells and Arabidopsis mesophyll protoplasts

**Fig. 5:**
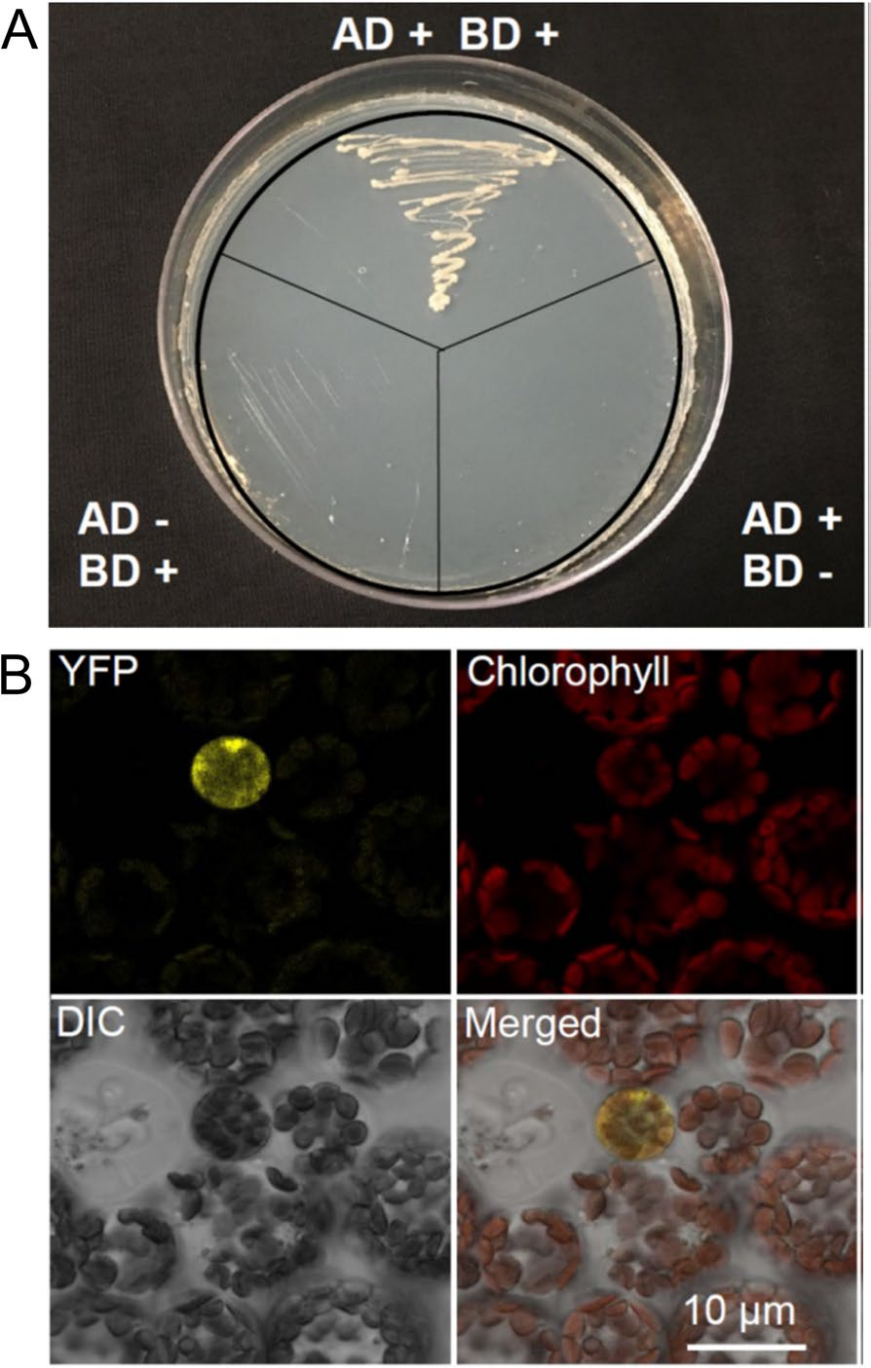
Physical interactions between SAG113 PP2C and SAG114 SnRK3.25 in both yeast and Arabidopsis leaf cells. **A** Yeast two-hybrid assay showing the interaction between SAG113 and SAG114.The coding sequences of SAG113 PP2C was fused with the GAL4 activation domain sequence of pGAD424 and the plasmid with the fusion was then transferred into PJ69-4α yeast cells. And the coding sequence of SAG114 SnRK3.25 was fused with the GAL4 binding domain of pGBT9 and the vector was then transferred into PJ69-4A yeast cells. the diploid cells generated via yeast mating were then streaked on SD/-Trp/-Leu/-His/-Ade plate. AD + , yeast cell containing pGAD424-SAG113 only; BD + , yeast cell harboring pGBT9-SAG114 only; AD-, yeast cell without pGAD424-SAG113; BD-, yeast cell without pGBT9-SAG114. **B** BiFC analysis of the interaction between SAG113-YFPN and SAG114-YFPC in the mesophyll protoplast of Arabidopsis. YFPN and YFPC represent N- and C-terminal half of the yellow fluorescent protein, respectively. The YFP panel, the yellow fluorescence imaged using the eYFP channel setting of Leica DM5500; Chlorophyll, chlorophyll autofluorescence; DIC, differential interference contrast image of the leaf mesophyll protoplasts. Merged, merged image of above images

The ABA-*AtNAP* transcription factor-*SAG113 PP2C* module had a pivotal role in regulating leaf senescence (Zhang and Gan 2012) and the SAG113 PP2C protein was localized in the Golgi apparatus (Zhang et al. 2012a). The Golgi apparatus-localization shown above raised the possibility that SAG114 might be a direct target or substrate of SAG113. We thus performed both yeast two-hybrid and bimolecular fluorescence complementation (BiFC) assays to test the possibility. In the yeast two-hybrid assay, cells harboring both pGBT9-SAG114 and pGAD424-SAG113 (AD + BD +) survived and propagated on a drop-out plate while the control cells (either AD- BD + or AD + BD-) did not (Fig. 5A), suggesting the physical interaction between the SAG113 PP2C and SAG114 in the yeast cells. The BiFC assay further revealed that both SAG113 PP2C and SAG114 proteins physically interact with each other in the mesophyll protoplasts of Arabidopsis (Fig. 5B).

### SAG114 was dephosphorylated by SAG113 PP2C in vitro and *in planta*

**Fig. 6:**
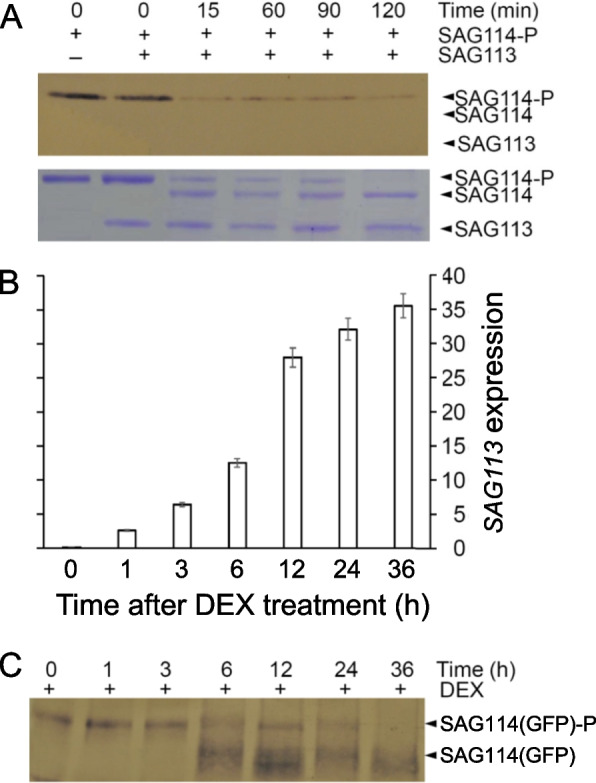
Dephosphorylation of SAG114 SnRK3.25 by SAG113 PP2C in vitro and *in planta*. **A** In vitro dephosphorylation assay. SAG113 and the phosphorylated SAG114 (SAG114-P) were co-incubated, separated on SDS-PAGE gel and stained with Coomassie Brilliant Blue (lower panel). The Phospho-Serine/Threonine-specific antibody was used to detect SAG114-P (upper panel). **B** qRT-PCR analysis of DEX-induced *SAG113* expression in non-senescing leaves of Arabidopsis that was constitutively expressing the SAG114-GFP fusion protein [SAG114(GFP)]. **C** Dephosphorylation of SAG114(GFP)-P by DEX-induced SAG113 *in planta*. Protein samples from leaves harvested at different time after DEX induction were separated on SDS gel, and the antibody against GFP was used to detect both SAG114(GFP)-P and its dephosphorylated form SAG114(GFP) (as well as its degraded form)

The physical interaction prompted us to hypothesize that SAG113 PP2C might dephosphorylate SAG114. To test the hypothesis, *E.coli* produced and phosphorylated SAG114 (SAG114-P) were incubated with *E. coli* produced SAG113 PP2C for various lengths of time. The samples were separated and subjected to Western blot analysis using Phospho-Threonine/Tyrosine Antibody. The antibody could detect proteins and peptides phosphorylated at threonine and tyrosine residues only (e.g., SAG114-P, but not SAG114) independent of the surrounding amino acid sequence (Invitrogen, USA). The amount of SAG114-P rapidly reduced and the amount of SAG114 (the dephosphorylated form) increased after the coincubation of the SAG113 and SAG114-P proteins (Fig. 6A), supporting that SAG113 PP2C could dephosphorylate SAG114 in vitro.

Next, we further investigated if SAG113 PP2C could dephosphorylate the SAG114 *in planta*. The SAG114-GFP fusion protein was constitutively expressed in Arabidopsis in which *SAG113 PP2C* could be chemically induced. Total proteins from non-senescent leaf samples harvested at different time points after the *SAG113* induction (Fig. 6B) were subjected to the Western blot analysis using antibody against GFP. The dephosphorylated form of the SAG114-GFP fusion protein started to accumulate 6 h after the DEX induction of *SAG113*, and the levels of the SAG114-GFP-P (phosphorylated form) was reduced (Fig. 6C). The fuzzy bands of the dephosphorylated form of SAG114-GFP could be due to the partial degradation of the fusion protein. These data suggested that SAG114 could be dephosphorylated by SAG113 PP2C *in planta*.

### *SAG114* was epistatic to *SAG113 PP2C*

**Fig. 7:**
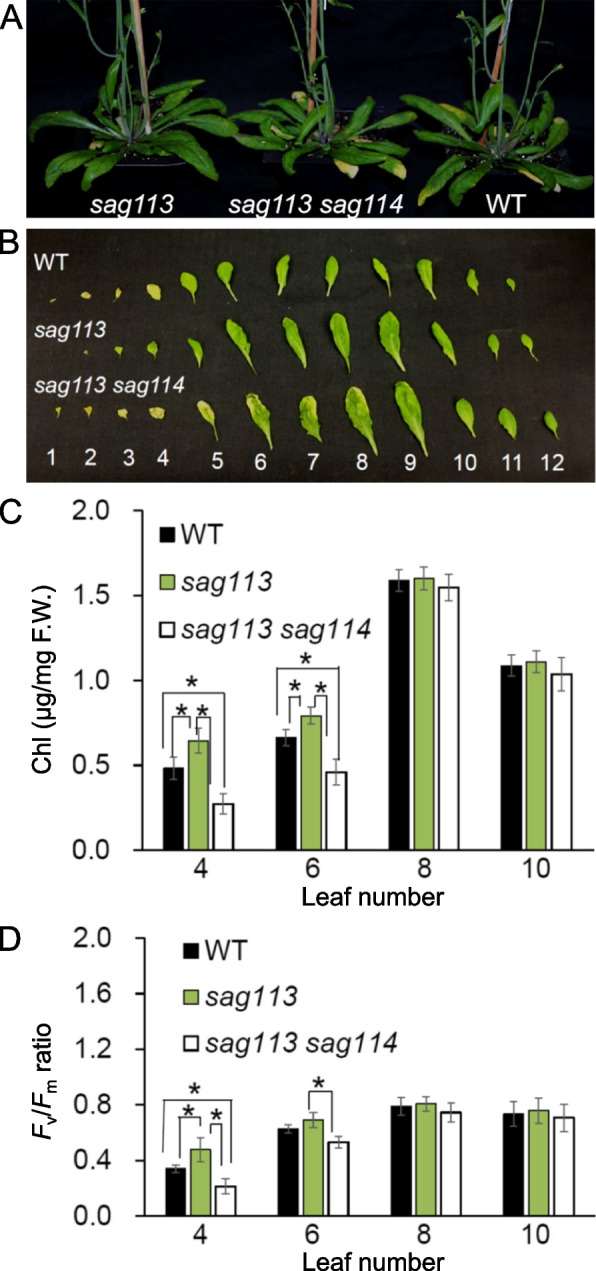
Epistasis of SAG114 SnRK3.25 to SAG113 PP2C. **A** Phenotype of age-matched *sag113*, *sag113 sag114* double mutant and WT. **B** Individual rosette leaves detached from the age-matched plants shown in A. The leaves were counted from bottom. The chlorophyll (Chl) contents (**C**) and the *F*v*/F*m ratios (**D**) in selected leaves shown in B. Mean values of four samples ± se are shown. Asterisks indicate significant differences between indicated plants (Student’s *t* test, *P* < 0.05)

The *sag113* null mutant showed a significant delay in leaf senescence (Zhang et al. 2012a) while *sag114* exhibited a precocious leaf senescence phenotype (Fig. 2). If SAG114 was a substrate of SAG113 PP2C as revealed above, the *sag113 sag114* double mutant would show an early leaf senescence phenotype, which was exactly what we observed (Fig. 7). The fact that *SAG114* was epistatic to *SAG113* further supported that *SAG114* was a direct target of *SAG113*.

## Discussion

**Fig. 8:**
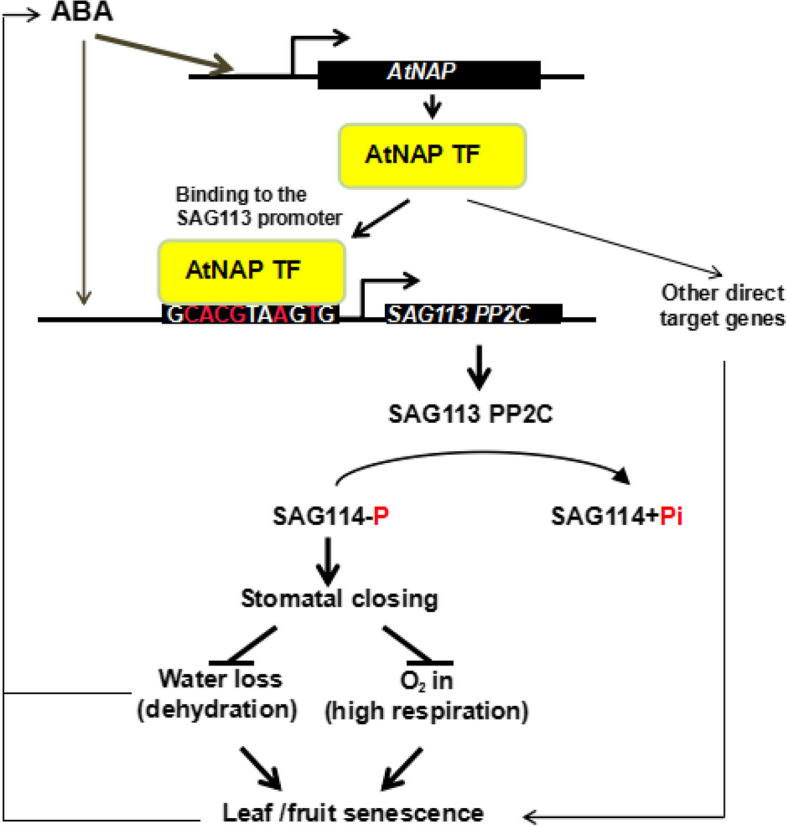
A working model of SAG114 SnRK3.25. SAG114 SnRK3.25 is the direct target of the ABA-AtNAP-SAG113 PP2C regulatory module (Zhang and Gan 2012). The phosphorylated SAG114 will promote stomatal closure, and the dephosphorylation by SAG113 PP2C will render SAG114 inactive. The figure is modified from Zhang and Gan (2012)

Leaf senescence is a programmed process with dehydration and nutrients reallocation, which can be induced by an array of internal (such as leaf age and hormones) and external factors. As a well-controlled genetic program (Gan 1995, 2022), many genes like those involved in photosynthesis are down-regulated while a set of senescence associated genes (*SAG*s) are up-regulated during leaf senescence. Approximately 10% of genes in the Arabidopsis genome or over 2,500 genes are up-regulated during leaf senescence. These *SAG*s may be involved in signal transduction, transcription, and nutrients reallocation (He et al. 2001; Guo et al. 2004; Cao et al. 2022). Individual *SAG*s have been characterized, and regulatory modules of some transcription factors (Guo et al. 2021; Cao et al. 2023) have been identified, for example, the ABA-AtNAP-SAG113 PP2C module was shown to prevent stomates from closing during leaf senescence (Zhang and Gan 2012). The underlying regulatory network is yet to be deciphered. This research revealed that SAG114 SnRK3.25 is a direct target of, and dephosphorylated by, the ABA-AtNAP-SAG113 PP2C module; the phosphorylated form of SAG114 promotes stomatal closure (Fig. 8).

There are three lines of evidence supporting that SAG114 and SAG113 PP2C interact with each other physically: (1) the yeast two-hybrid experiment (Fig. 5A), an artificial genetic system for detecting and assessing protein–protein interactions; (2) the BiFC assay (Fig. 5B), a method used to directly visualize protein–protein interaction in vivo using live-cell imaging; and (3) such an interaction between SAG113 PP2C and SAG114 is reinforced by their identical subcellular localization: SAG113 protein was found in the Golgi apparatus (Zhang et al. 2012a), and SAG114 protein was also localized in the Golgi apparatus (Fig. 4).

*SAG113* encodes a protein phosphatase 2C (Zhang et al. 2012a), and SAG114 appears to be the direct target or substrate of the SAG113 PP2C, which is supported by the facts that SAG113 PP2C is able to remove the phosphate group from SAG114 both in vitro (Fig. 6A) and *in planta* (Fig. 6B). Protein phosphorylation is critical in signaling and/or protein function, and the removal of the phosphate group or dephosphorylation often renders the target protein non-functional (Zhou et al. 2009).

*SAG114* encodes an apparent protein kinase named SnRK3.25 (Fig. S1). It is expressed specifically in senescing leaves and associated stomates (Fig. 1). It appears to be expressed in senescing flowers as well (http://bar.utoronto.ca/efp/cgi-bin/efpWeb.cgi?primaryGene=AT5G25110&modeInput=Absolute). When knocked out, *sag114* displays a precocious leaf senescence phenotype (Fig. 2) with significantly larger stomatal aperture and faster water loss (Fig. 3, Fig S2), suggesting that phosphorylated SAG114 SnRK3.25 promotes stomatal closure (Fig. 8), and the dephosphorylation of SAG114 by SAG113 PP2C will prevent the stomates from closing. The SAG114 SnRK3.25 acting immediate down stream of SAG113 PP2C is supported by the fact that *sag114* is epistatic to *sag113* (Fig. 7). In addition to the important role in leaf (and potentially flower) senescence, *SAG114* is involved in responses to hypoxia and salt stress (Amarasinghe et al. 2016; Tagliani et al. 2020).

There are two biological questions concerning SAG114 SnRK3.25 that are yet to be addressed: (1) how is SAG114 SnRK3.25 phosphorylated? Overexpressed SAG114-GFP fusion protein appeared to be in the phosphorylated form only (Fig. 6C). It could be self-phosphorylated. It also could be phosphorylated by another protein kinase(s) such as MPK6 (Zhou et al. 2009). (2) What is/are the immediate target(s) or substrate(s) of SAG114 SnRK3.25? Phosphoproteomics analysis (Yan et al. 2022) involving various mutants such as *sag114* and *mpk6* may be used to investigate into these questions.

## Material and methods

### Plant materials and growth conditions

Arabidopsis (*Arabidopsis thaliana*) ecotype Columbia was used in the study. The *sag114* knockout mutants, transgenic lines and the related *SAG113*-inducible expression lines are all in Columbia background (Zhang and Gan 2012). Two SALK T-DNA insertion lines (SALK_06012 and SALK_079011) were obtained from Arabidopsis Biological Resource Center. All seeds were sterilized in 70% ethanol containing 0.01% Triton X-100 for three times, and then sown on petri dishes containing Murashige and Skoog salts with 0.7% w/v phytoagar (Sigma, USA) and appropriate antibiotics. The dishes were kept at 4℃ for 24 h and then moved to a growth chamber at 22℃ with 60% relative humidity under continuous light (110 µmol m^−1^ s^−1^) from a mixture of fluorescent and incandescent bulbs. Approximately 8d after germination (DAG), seedlings were transplanted to Cornell mix soils (3:2:1 peat moss: vermiculite: perlite, v/v/v) and grew in a growth chamber. The mutants, transgenic plants, and wild type were grown side by side.

### Plasmid construction

The coding region of *SAG114* used in this research was amplified using primers G4692 (5’-CCCGGGCATGGGATCCAAACTTAAACT-3’, the underlined section is an engineered SmaI site) and G4693 (5’-CTGCAGTCTTAGCAGTCACTACCAGAATTTTC-3’, the underlined section is an engineered Pstl site) on the template of cDNA from senescence leaves. the coding region of SAG113 was amplified using primers G4690 (5’- CCCGGGCATGGCTGAGATTTGTTAC-3’, the underlined section is an engineered SmaI site) and G4691 (5’-CTGCAGAACTACGTGTCTCGTCGTAGAT-3’, the underlined is an engineered Pstl site) on the template of cDNA from senescence leaves. pGEM-SAG114 and pGEM-SAG113 were constructed for yeast two-hybrid assay, and pBJ36-SPYNE-SAG113 and pBJ36-SPYCE-SAG114 for bimolecular florescence complementation. For the SAG114 subcellular study, the *SAG114* coding region was PCR amplified using primers G3661 (5’-GTCGACGGATGGGATCCAAACTTAAACTTTAC-3’, the underlined section is an engineered SalI site) and G332 (5’-CCGCGGTAGCAGTCACTACCAGAATTTTCATC-3’, the underlined section is KpnI site) and was cloned into pGEM-T easy vector (Promega, USA). The insert was sequenced (to ensure that no mutations were introduced), cut with SalI and PstI, and subcloned into the eGFP vector pSAT6-GFP-N1(Zhang et al. 2012a) to form pGL4120. The *SAG114-GFP* fusion was released from the plasmid with a restriction enzyme called PI-P*sp*I and subcloned into pPZP-RCS vector to form the SAG114-GFP fusion protein expression vector pGL4121.

For P_*SAG114*_:*GUS* construct, the *SAG114* promoter was PCR amplified using primers SAG114-P1 (5'-AATTTTGGAGGTAACACTTT-3’) and SAG114-P2 (5’-GTGTATATACAGAAGTAGAA-3’) and was cloned into pMD18-T vector (TaKaRa, Japan). The insert was sequenced (to ensure that no mutations were introduced), cut with BamHI and HindIII, and subcloned into the pCAMBIA1301 to generate the P_*SAG114*_:*GUS* vector.

### Histochemical GUS staining

The transgenic Arabidopsis plants containing the P_*SAG114*_:*GUS* were used for β-glucuronidase (GUS) histochemical staining. Seedlings of the P_*SAG114*_:*GUS* transgenic mature plants were used for the location of *SAG114* expression. GUS histochemical staining was performed as reported previously (He et al. 2001), with some modifications: fixation was done by immersion of the tissues in an ice-chilled 90% acetone (v/v) bath, followed by incubation for 20 min on ice and rinsing three times with the solution [100 mM sodium phosphate buffer (pH 7), 10 mM EDTA, and 0.1% (v/v) Triton X-100]. Then, the tissue was incubated in staining solution [1 mM 5-bromo-4-chloro-3-indolyl-beta-D-glucuronic acid, cyclohexylammonium salt (X-Gluc, Thermo Scientific), 100 mM sodium phosphate buffer (pH 7), 10 mM EDTA, and 0.1% (v/v) Triton X-100], 12–16 h at 37℃. After incubation, the tissues were cleared with 50%, 60%, 70%, 80%, 90% (v/v) ethanol gradually. The GUS-stained tissues were imaged with a stereomicroscope (DFC295, Leica Microsystems Ltd., Germany).

### Phylogenetic analyses

The phylogenetic analyses were carried out according to the methods described by Zhang et al. (2012a). Amino acid sequences were extracted form TAIR database (https://www.arabidopsis.org). A neighbor-joining tree was built using MEGA (version 7.0) adopting Poisson correction distance and was presented using radiation treeview. Support for the obtained tree was assessed using the bootstrap method with 1000 replicates.

### Yeast two-hybrid assay

Full-length cDNA sequences of *SAG113* and *SAG114* coding region were cloned into pGEM-T (Promega, USA) to form pGEM-SAG114 and pGEM-SAG113. The coding sequence was then released from pGEM-SAG114 and pGEM-SAG113 with SmaI and PstI, and was subcloned into SmaI and PstI sites of pGBT9 and pGAD424 (New England Biolabs, USA) to form pGBT9-SAG114 and pGAD424-SAG113 vectors, respectively. All constructs were confirmed by sequencing and then transformed into yeast strain PJ69-4α and PJ69-4A following standard transformation techniques. Transformants were grown on proper drop-out plates for selection, mating, and further selection (Zhao et al. 2015).

### Bimolecular florescence complementation (BiFC) and transient expression

To generate BiFC constructs, the full-length cDNA sequences of *SAG113* and *SAG114* were directly cloned into pBJ36-SPYNE (YFP N-terminal portion) and pBJ36-SPYCE (YFP C-terminal portion) vector by Gibson DNA assembling (Guan et al. 2017). Each cassette was then cut and cloned into the NotI site of pGreenII0179 (SPYCE cassettes) or pGreenII0229 (SPYNE cassettes). And transient expression was conducted following Guan’s method (2017). All the constructs were transformed into the *Agrobacterium tumefaciens* AGL-0 strain in BiFC experiment. Agrobacterium solution was adjusted to OD600 nm = 0.5 and then equally mixed before Agroinfiltration. Arabidopsis protoplasts were used for co-expression studies as previously described. The fluorescence signal was detected 2 d after infiltration, using microscope to acquire fluorescence image. Yellow fluorescent protein (YFP) imaging was performed at an excitation wavelength of 488 nm (Hemerka et al. 2009).

### Fluorescence microscopy analyses

The fluorescence microscopy assays were performed as described (Zhang et al. 2012a). Stable expression of GFP control, SAG114-GFP and ERH1-DsRED (Zhang et al. 2012a) fusion proteins in T_2_ generation were examined and photographed using an SP5 laser scanning confocal microscope (Leica DM5500, USA). The GFP signal was acquired using the eGFP channel setting while RFP signal was acquired using the DsRED channel.

### Chlorophyll assay and *F*_v_/*F*_m_ analysis

Chlorophyll was extracted and quantified as described previously (Wang et al. 2022). Total fluorescence in leaves was measured using a portable modulated chlorophyll fluorometer (model OS1-FL) according to the manufacturer’s instructions (Opti-Sciences, Tyngsboro, MA). The variable and maximal fluorescence (*F*_v_/*F*_m_) of individual leaves was quantified directly using the fluorometer’s module 9 program (He and Gan 2002).

### Measurement of water loss and stomatal aperture assays

Both mature leaves and early senescing leaves from *sag114* and WT were sampled and the fresh weight was recorded as W_0_. Subsequently, each sample was dried at room temperature for 120 min. The leaves of different lines were weighed every 30 min as W_1_. the ratio of water loss (RWL) was calculated as RWL = (W_0_-W_1_) / W_0_ × 100% (Dong et al. 2012).

For stomatal aperture assay, leaves of WT and mutants at the non-senescent and early senescent stages were applied with colorless nail polish. Ten minutes later, the polish on the leaf epidermis was peeled off, and the stomatal aperture was examined under a microscope with a CCD camera (Zeiss, http://www.zeiss.com/).

### Transcript analysis

RNA extraction were performed according to Zhang and Gan (Zhang and Gan 2012). First-strand cDNA was synthesized from 3 µg of total RNA (treated with DNase) at 42 °C with MV-reverse transcriptase (Promega, USA). For each reverse transcription-PCR, 1 µL of each diluted sample was used as a template in a 20-µL reaction following the standard methods. For real-time PCR, all PCR reactions were performed on a Bio-Rad IQ-5 thermocycler with 40 cycles and an annealing temperature of 55 °C. Cycle threshold values were determined by the IQ-5 Bio-Rad software assuming 100% primer efficiency. The primers G3221 (5’-CGGGTGGTCGTGTTATCTACTG-3’) and G3222 (5’-CCTCCGGTCTGCTGATTACATAC-3’) were used for *SAG113* gene qPCR assay. Primers G4700 (5’-AATGGGGAAGCTTGAAGGGA-3’) and G4701 (5’- TATCTCCCGCCGACTTACAC-3’) were used for *SAG114* gene qPCR assay. The primers G3053 (5’-AGTGGTCGTACAACCGGTATTGT-3’) and G3054 (5’-GATGGCATGAGGAAGAGAGAAAC-3’) were used for *Action 2* gene qPCR assay. Gene expression was normalized relative to the expression of *Actin 2*. Three repetitions were performed for each combination of cDNA samples and primer pairs.

### In vitro dephosphorylation assay

Twenty-four hours after *E. coli* strain TB1 cells were transiently transfected with vectors encoding SAG113 and SAG114, the cells were washed with ice-cold PBS and lysed with HEST buffer [50 mM HEPES (pH7.2), 5 mM EDTA, 0.25 M sucrose, and 1% Triton X-100] containing protease and serine phosphatase inhibitors, 1 mM PMSF, 1 mM NaF, and protease inhibitor mixture (Sigma, USA). Samples were incubated on ice for 30 min, and centrifuged (14,000 × g) for 10 min at 4 °C to remove insoluble material. The fusion proteins were purified by amylose-affinity chromatography (New England Biolabs, USA) and were quantified using Bio-Rad Protein assay reagent (Bio-Rad laboratories, USA). Phosphorylated SAG114 proteins (SAG114-P) were obtained by treatment with MPK6 using the method described by Zhou et al (2009). The SAG113 lysates and SAG114-P solution were then mixed in equal volume and incubated at 35 °C with agitation for the indicated time periods. Phosphoserine/threonine antibody (Invitrogen, USA) was used to detect the SAG114-P levels by immunoblot analyses according to the manufacturer’s instruction.

### *In planta* dephosphorylation assay

The dexamethasone (DEX)-inducible *SAG113* overexpression homozygous line (Zhang and Gan 2012) was crossed with SAG114-GFP expression homozygous line. The 20-day-old F1 plants were treated with 10 mM DEX and leaves were sampled at different time points. Proteins were extracted and immunoblots were used to detect the phosphorylated and dephosphorylated SAG114-GFP fusion protein levels with the phosphoserine/threonine antibody (Invitrogen, USA) and GFP antibody (Invitrogen, USA), respectively. Briefly, leaf sample (250 mg) was lysed in 1 mL of TBST buffer (0.1% Tween-20, 100 mM Tris–HCl, and 150 mM NaCl, pH 7.5). After centrifugation at 12,000 g for 10 min, the supernatant was mixed with an equal volume of loading buffer (100 mM Tris- HCl, 5 mM DTT, 4% SDS, 0.01% bromophenol blue, and 30% glycerol, pH 6.8), separated by 12% SDS-PAGE, and transferred onto an Immobilon-PPSQP transfer membrane (polyvinylidene fluoride (PVDF) type; Millipore) using a Bio-Rad mini transfer cell. The membrane blots were incubated in blocking buffer (5% milk, 0.1% Tween-20, 0.1% Triton X-100, 100 mM Tris–HCl and 150 mM NaCl, pH 7.5) for 2 h at room temperature, washed twice with TBST buffer and incubated with related antibodies (1:10000 dilution) for 20 h at 4 °C. After two rinses with TBST, the blots were incubated in 1:10000-diluted secondary antibody solution (affinity-purified HRP-conjugated Affinipure goat anti-rabbit IgG (H + L), for 2 h at room temperature and washed twice with TBST. The blots were incubated in the 3,3’-diaminobenzidine color development substrate system (Sigma, USA) according to the manufacturer’s instructions.

### Supplementary Information

**Additional file 1: Supplemental Figure S1.** SAG114 (AT5G25110) SnRK3.25 is a typical protein of SnRK family. **Supplement Figure S2.** Stomatal aperture in *sag114* is larger than that of WT in both mature leaves and senescing leaves.

## Data Availability

The data and materials will be available upon reasonable request.

## Abbreviations

ABA: Abscisic acid
AMPK: Adenosine monophosphate-activated protein kinase
BiFC: Bimolecular fluorescence complementation
CBL: Calcineurin B-like protein
CDPK: Calcium-dependent protein kinase
CIPK: Calcineurin β-like interacting protein kinase
DEX: Dexamethasone
DsRed: Discosoma brilliantly red fluorescent protein
GFP: Green fluorescent protein
GUS: β-Glucuronidase
MAPK: Mitogen-activated protein kinase
NAF: Asn-Ala-Phe
NAP: NAC-LIKE, Activated BY AP3/P1
RWL: Ratio of water loss
PP2C: Protein phosphatase 2C
SAG: Senescence-associated gene
SnRK: Sucrose nonfermenting 1-related kinase
WT: Wild type
