# Supplementary material for: The ABA–AtNAP–SAG113 PP2C module regulates leaf senescence by dephoshorylating SAG114 SnRK3.25 in Arabidopsis
Source: Mol Hortic. 2023 Oct 30;3:22. doi: 10.1186/s43897-023-00072-1 (PMC10614403; doi:10.1186/s43897-023-00072-1)
Supplement: Supplementary file 1 — Additional file 1: Supplemental Figure S1. SAG114 (AT5G25110) SnRK3.25 is a typical protein of SnRK family. Supplement Figure S2. Stomatal aperture in sag114 is larger than that of WT in both mature leaves and senescing leaves. [file 43897_2023_72_MOESM1_ESM.pptx]

## Slide 1
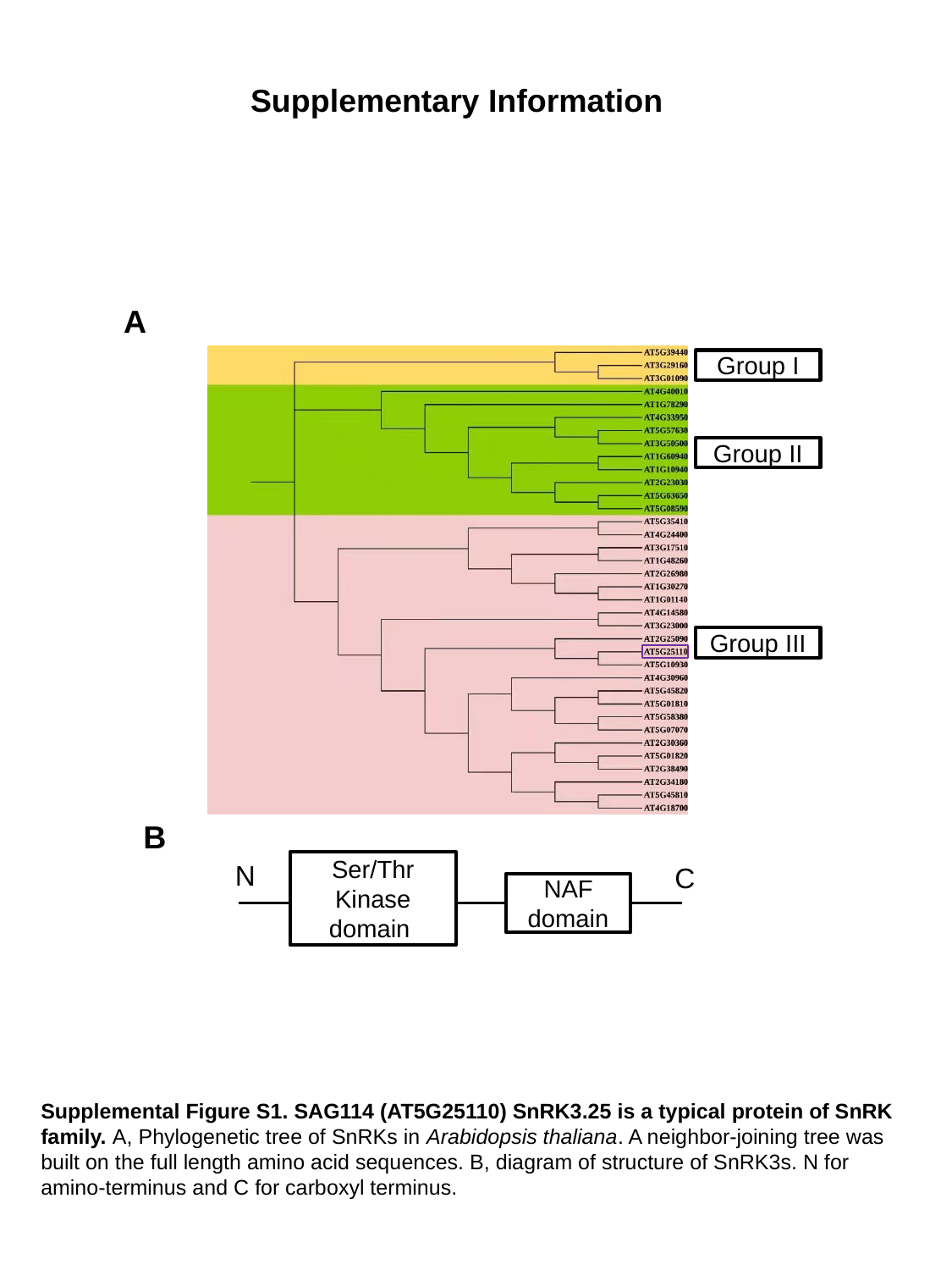

Supplementary Information
A
Group I
Group II
Group III
B
N
C
NAF
domain
Ser/Thr
Kinase domain
Supplemental Figure S1. SAG114 (AT5G25110) SnRK3.25 is a typical protein of SnRK family. A, Phylogenetic tree of SnRKs in Arabidopsis thaliana. A neighbor-joining tree was built on the full length amino acid sequences. B, diagram of structure of SnRK3s. N for amino-terminus and C for carboxyl terminus.

## Slide 2
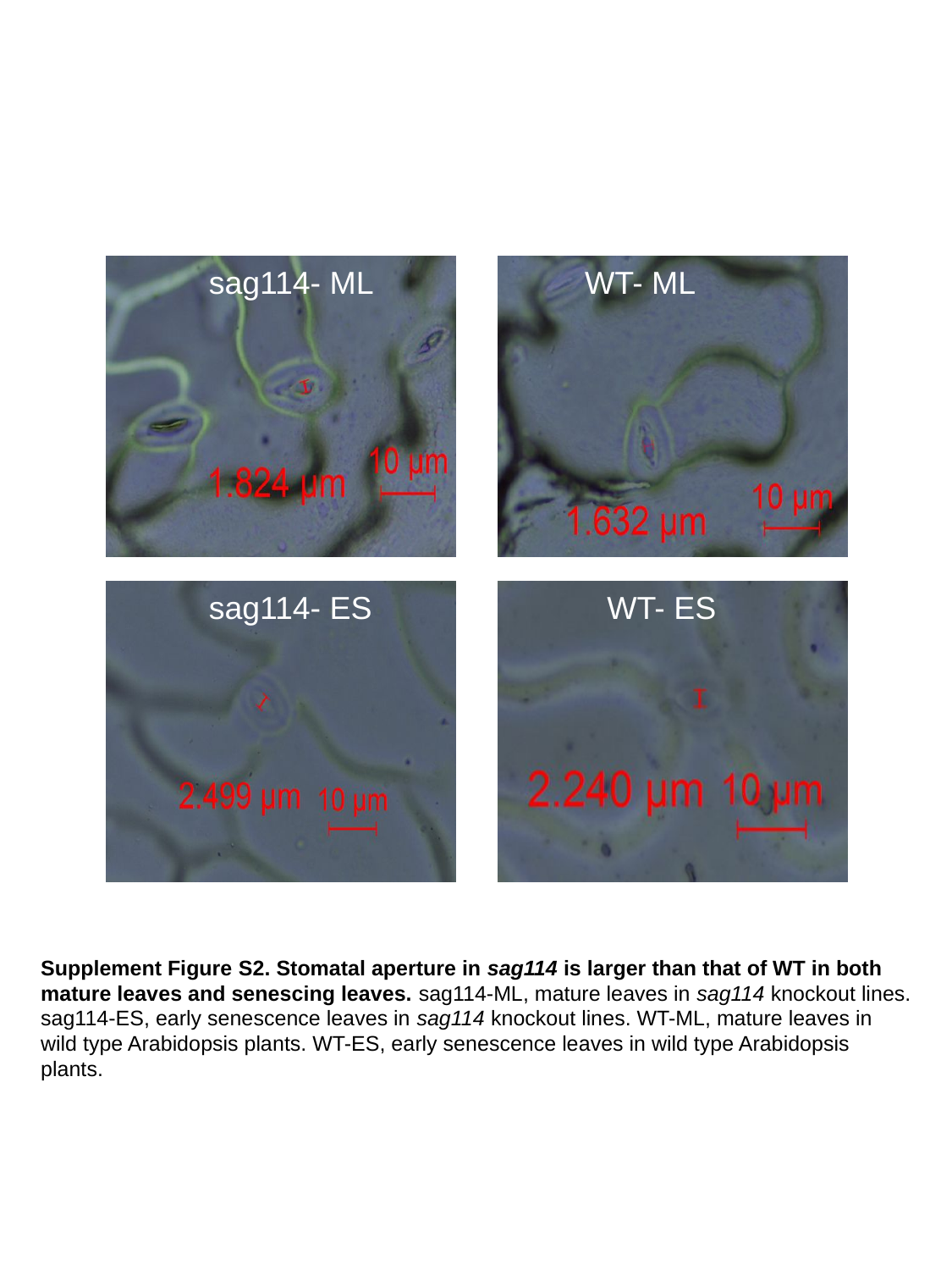

sag114- ML
WT- ML
sag114- ES
WT- ES
Supplement Figure S2. Stomatal aperture in sag114 is larger than that of WT in both mature leaves and senescing leaves. sag114-ML, mature leaves in sag114 knockout lines. sag114-ES, early senescence leaves in sag114 knockout lines. WT-ML, mature leaves in wild type Arabidopsis plants. WT-ES, early senescence leaves in wild type Arabidopsis plants.
